# Field data on sea ice restoration by artificial flooding

**DOI:** 10.1016/j.dib.2024.111117

**Published:** 2024-11-12

**Authors:** Tim C. Hammer, Aleksey Shestov, Laurina Leuntje van Dijke, Fonger Ypma, Tom Meijeraan, Hayo Hendrikse

**Affiliations:** a Faculty of Civil Engineering and Geosciences, Department of Hydraulic Engineering, Delft University of Technology, Delft 2628 CN, the Netherlands; b Arctic Technology Department, The University Centre in Svalbard, P.O. Box 156N-9171, Longyearbyen, Norway; c Arctic Reflections, Paardenmarkt 1, 2611 PA Delft, the Netherlands

**Keywords:** Snow, Albedo, Snow ice, Melting, Radiation

## Abstract

A field campaign in the Vallunden lagoon in the Van Mijenfjorden on Spitsbergen was conducted to gather data on sea ice restoration by artificial flooding. Sea ice thickening was initiated by pumping sea water from below the first-year sea ice onto the surface without removing the covering snow layer. Part of the data was collected by four thermistor strings, two radiation sensors, and one anemometer. All measurement systems were left in the field until recovery of the floating systems in summer. Data provided by the measurement devices were received remotely to gather data before, during, and after the flooding phase (including the melting for as long as the sensors were sending data). Furthermore, coring systems were used to extract 88 ice cores for analysis of temperature, density and bulk salinity profiles along the full length of the ice cores before, during and within four days after flooding. The data set can be used to investigate physical processes involved in the ice growth before, during and after flooding. The data can be used to understand the development, growth and melting of snow ice. The radiation data can be used to analyze the (reflected) radiation of the initial, flooded and melting ice. Data gathered during the melting can be used to investigate the melting of thickened sea ice with different initial conditions prior to the onset of melting. Data on bulk salinity can be used to investigate short-term salt migration. Combing the different insights, growth- and melting models of sea ice including snow and snow ice can be validated. The understanding of melt-water drainage events could be improved and flow models for simulation of artificial flooding of snow-covered first-year sea ice could be further developed using the data.

Specifications Table

**Table:** 

Subject	Earth-Surface Processes Alternatively: Atmospheric Science Alternatively: Environmental Engineering
Specific subject area	Artificial flooding of snow-covered first-year sea ice and associated geophysical freezing and melting processes.
Type of data	Raw (.csv, .xlsx), Postprocessed (.mat), Video (.mp4), Images (.zip, .jpg).
Data collection	Field data were collected in 2024 at Vallunden lagoon on Spitsbergen. The test sites were flooded in the period between April 10 and April 15. The measurement stations collected continuous data from March 21 to June 24 on one site and from April 10 to June 24 on three further sites. 88 ice cores were taken in the period between April 11 to April 15. The data collection involved various instruments. Thermistor strings (Snow and Ice Mass Balance Apparatus (SIMBA) – SAMS Enterprise) were used to monitor temperature, with one installed at each site. Radiometers (Apogee Instruments SN-500 Net Radiometer – METER Group), consisting of up- and down-looking pyranometers (SP-510-SS and SP-610-SS) and pyrgeometers (SL-510-SS and SL-610-SS), were installed at Site A (i.e., reference site) and Site B (i.e, flooded site) to measure radiative fluxes. Wind speed and direction were measured using an ultrasonic anemometer (ATMOS 22 Ultrasonic Anemometer – METER Group) at Site B. All instruments were connected to a ZL6 Advanced Cloud Data Logger (METER Group) for continuous data recording during the flooding, freezing, and melting period. Flooding tests were conducted using an EMV 160 pump (Erikssons Mekaniska Verkstad AB) for controlled water discharge. In-situ ice coring was performed using Kovacs Mark II and Mark III coring systems (both Kovacs Enterprise) to extract samples for analysis. Ice cores were analyzed for temperature, bulk salinity, and density at 5 cm intervals for temperature and 10 cm intervals for salinity and density using a TFX 410 Core Thermometer with a fixed Pt 1000 Probe (Xylem Analytics Germany Sales GmbH & Co. KG), and a GMH 3431 Conductivity Meter (Senseca Germany GmbH), alongside a KERN KB-2000 Precision Balance (KERN & SOHN GmbH). Documentation of field activities was carried out using a Tikee 3 PRO+ camera (Two Sony Exmor R 16 Mpx image sensors) and a DJI Mavic 2 Pro drone (DJI Sky City) to capture photographic records of the sites and procedures. The provided data are raw data. The only postprocessed data (i.e., .mat) were sorted by date. No data have been excluded.
Data source location	Vallunden lagoon Spitsbergen Norway
Data accessibility	Repository name: 4TU.ResearchData Data identification number: *(*10.4121/dae61d9b-d079-4959-868e-591b7de1b7f6*)* Direct URL to data: https://data.4tu.nl/datasets/dae61d9b-d079-4959-868e-591b7de1b7f6
Related research article	Related research article: None.

## 1 Value of the Data

• These data allow to study the effect of flooding snow-covered first-year sea ice on the (snow) ice growth and melting processes of the ice sheet.
• Ice models addressing the growth and melting of first-year sea ice including snow ice can be validated.
• The long- and short-wave radiation variation during the flooding, and thickening of snow-covered first-year sea ice, and melting of snow ice-covered first-year sea ice can be investigated.
• The dataset may be used as reference dataset for further analysis of ice property development in the face of climate change (i.e. more frequent flooding scenarios).
• Researchers, policymakers, engineers and the social and public sector leaders may benefit from this dataset as it may help to make informed decision on the use of ice restoration as, for example, a solar radiation method or climate adaptation method.

## 2 Background

Engineers aim to thicken first-year sea ice in winter through artificial flooding, potentially increasing the time ice reflects sunlight during the melting season. One theoretical study investigated the use of wind-powered flood pumps to artificially flood existing sea ice [1]. Another study assessed the impact of sea ice thickening on the climate by numerical modeling [2]. Another study, incorporating the flooding of the snow layer, found that the greatest increase in sea ice volume and area occurs when flooding takes place in September and October in the Arctic [3]. However, the scalability of local geoengineering on the global climate is debated [2], with suggestions that a fourfold local reduction in solar radiation is necessary to preserve summer Arctic sea ice [4].

It remains unclear whether observations from natural flooding events—caused by lateral seawater intrusion [5], upward brine percolation [6], melting of upper snow layers [7], or liquid precipitation [8]—are applicable to artificial flooding scenarios. Factors, important for the ice-climate modeling, such as time- and depth-dependent salinity, density, thermal conductivity of snow and ice, surface albedo, and the formation of snow ice by mechanisms other than gravitational snow flooding [8], are supposed to be of great importance when artificial flooding of sea ice is considered.

After all, climate predictions based on artificial flooding are focus of debate, especially as the flooding of the snow-covered sea ice is not well understood.

This study presents field experiments designed to gather data on the physical processes involved during the artificial flooding of snow-covered ice, such as the formation of snow ice and the melting of thickened sea ice. Furthermore, the data may help to determine whether sea ice restoration by artificial flooding prolongs the longevity of sea ice during the summer melt for the given initial and boundary conditions.

## 3 Data Description

**Fig. 1:**
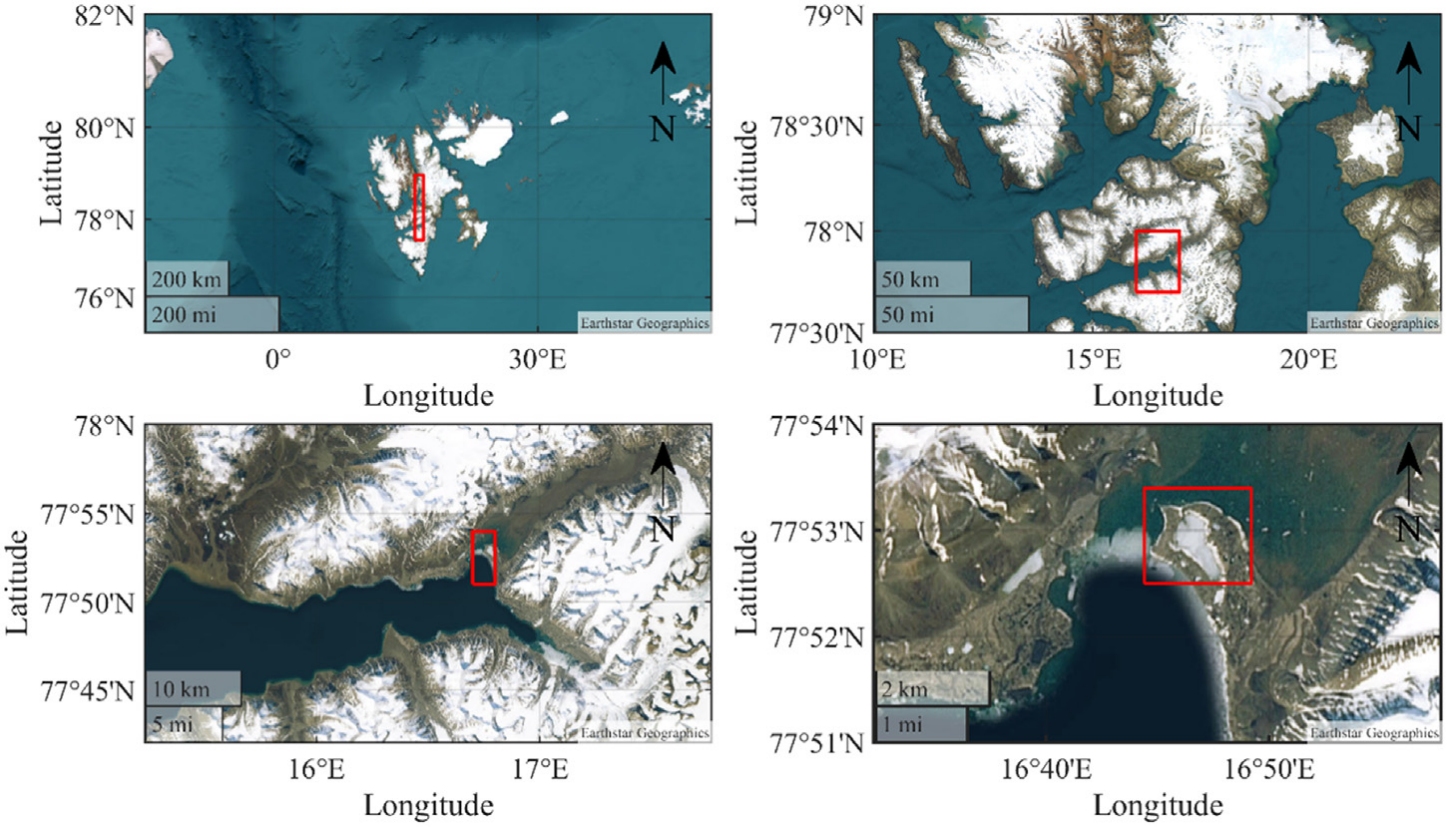
The field campaign was conducted in the Vallunden lagoon which is connected through a small water inlet with the Van Mijenfjord on Spitsbergen.

The data were collected in the Vallunden lagoon in 2024 on Svalbard (see Fig. 1) [9]. The data description is separated into three subsections. The first section is describing the data collected by installed measurement stations in the ice. The second section is describing the data gathered by coring ice samples. The third section is describing the footage taken and provided.

### 3.1 Measurement stations

**Fig. 2:**
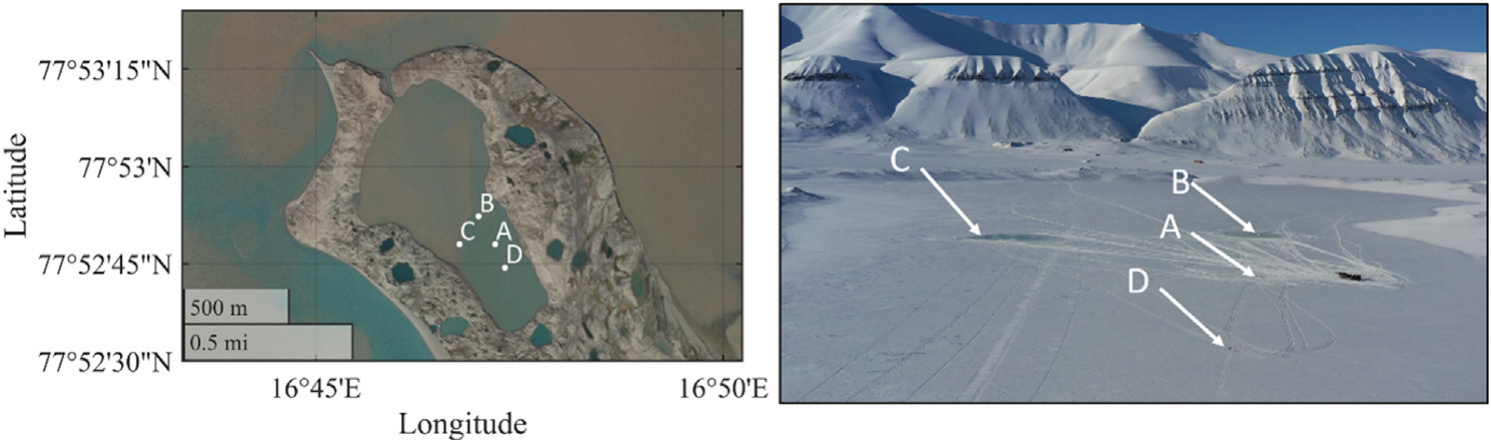
Left: Close up on the Vallunden lagoon. Right: Drone footage during the field campaign. Both pictures show the Sites A, B, C, and D.

In total four measurement stations have been employed (see Fig. 2). Each station was in the center of a test site. The test site names, coordinates, and measurement systems for each measurement station are given in Table 1.

**Table 1:** Description of the four measurement stations.

Names	Center coordinates	Measurement systems and data coverage interval
Site A	77.8800°N 16.7867°E	Thermistor string, (radiometer)
		10.04.2024-24.06.2024 (18.06.2024)
Site B	77.8812°N 16.7833°E	Thermistor string, (radiometer, anemometer)
		21.03.2024-24.06.2024 (19.06.2024)
Site C	77.8800°N 16.7794°E	Thermistor string
		10.04.2024-24.06.2024
Site D	77.8790°N 16.7887°E	Thermistor string
		10.04.2024-24.06.2024

The thermistor string measured the vertical temperature profile over a total length of roughly 4.80 m with a spatial resolution of 2 cm along the string. The sampling rate was set to 15 min during the period of flooding, and freezing of the flooded layer (i.e., 11.04.2024 – 23.04.2024). In the period after (i.e., 23.04. 2024 – 24.06.2024) the sampling frequency was set to 2 hrs. In addition to regular temperature measurements, the thermistor string also performs heating cycles every 24 hrs. During each cycle, the temperature sensors get heated by a specific heating element within two specific time intervals. The heating is performed to help identifying the medium surrounding the thermistor string (i.e., air, snow, water) by approximating a thermal diffusivity.

The radiometer installed on Site A and B measured the incident and reflected short wave radiation with a pyranometer, and the incident and emitted long wave radiation with a pyrgeometer on each side of the sensor, respectively. The anemometer used ultrasonic waves for determination of the wind speed, and therefore also internally measured the air temperature. Both the radiometer and the anemometer used a sampling rate of 5 min during the measurement period on Site A (i.e., 11.04.2024 – 18.06.2024) and Site B (i.e., 21.03.2024 – 19.06.2024). The data were sent to the data logger which uploaded the data in near-real-time to a ZENTRA cloud providing remote access to the data.

Data measured by the thermistor strings are provided in a separate table for each test site (Site A^1^, B^2^, C^3^, and D^4^), which can be found in the data repository [9]. The content of these tables is described in the accompanying *readme.pdf* file in the data repository. Data obtained from the radiometer and anemometer are provided in separate *.mat* files for Site B^5^ and Site A^6^. The files can be found in the data repository respectively. In difference to the raw data, the data provided in the *.mat* files has been sorted by the date of recording. The content of both *.mat* files is described in Table 4 and Table 5 of the accompanying *readme.pdf* file in the data repository.

**Fig. 3:**
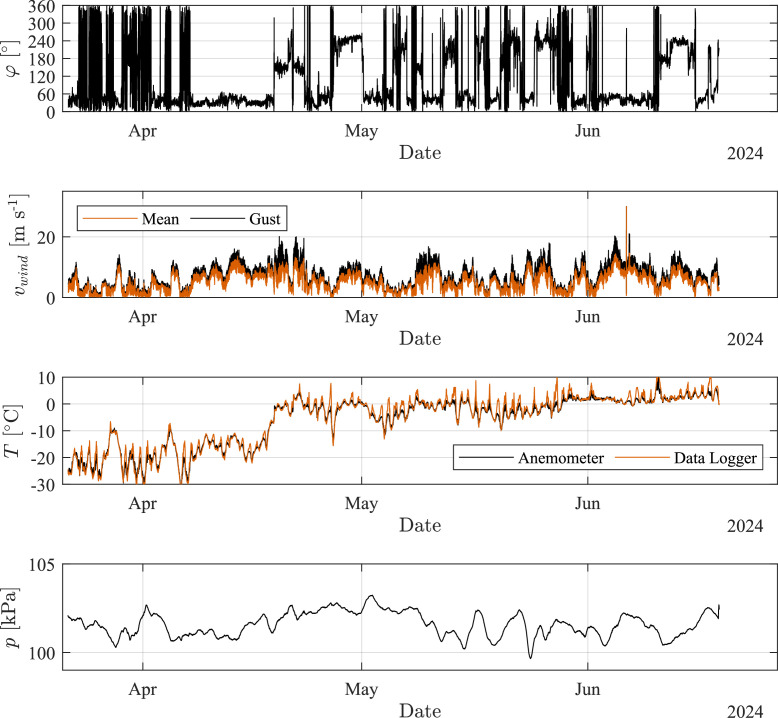
Visualization of environmental data measured on Site B.

**Fig. 4:**
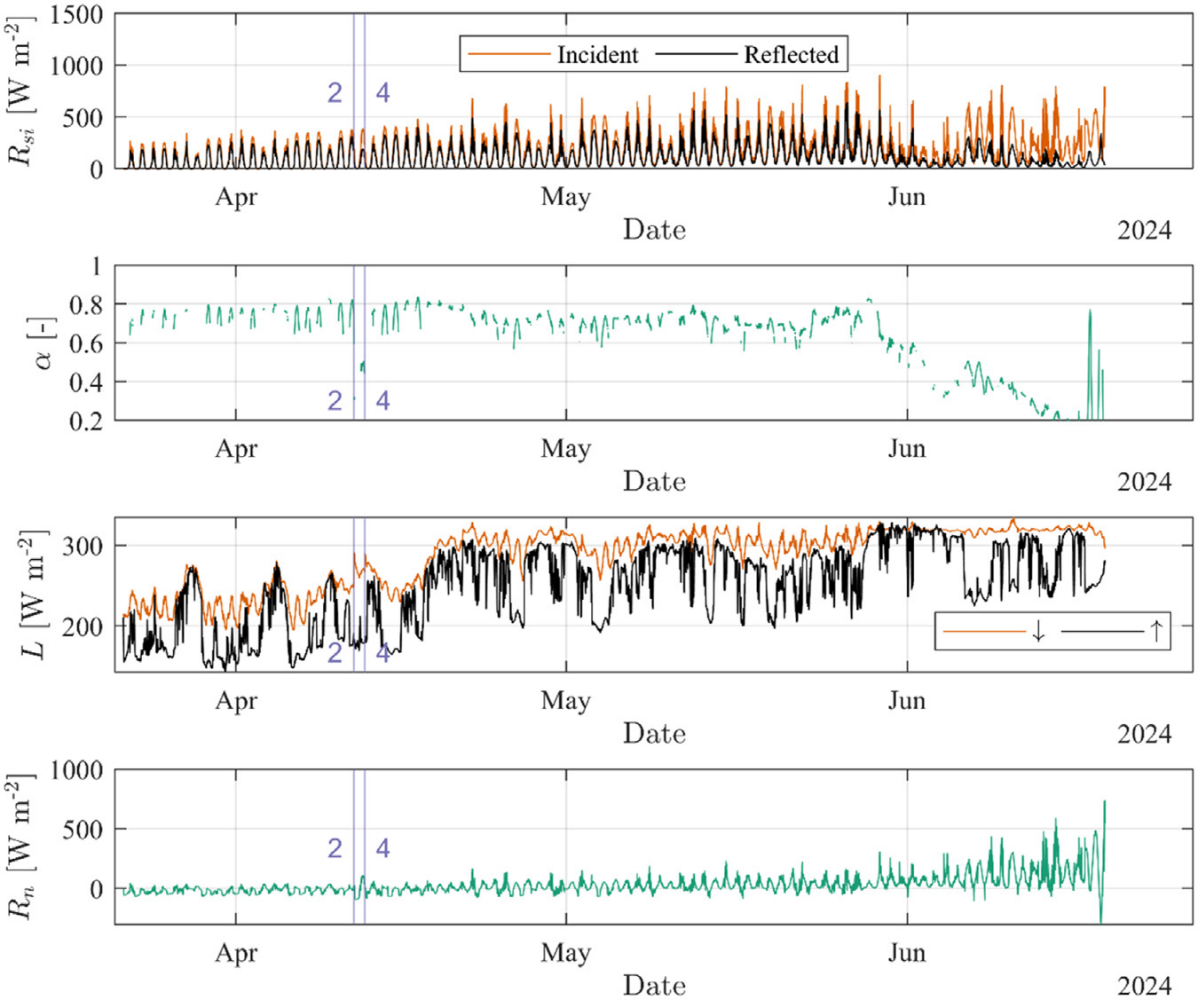
Radiation measurement from Site B. The two flooding events #2 and #4 are indicated in time.

Fig. 3 shows an example on how the data gathered on Site B can be used to visualize the wind direction φ (as defined in Fig. 4), the mean and gust wind speed $v_{wind}$, the air temperature *T*, and the atmospheric pressure *p* as measured by the anemometer and data logger respectively.

Similarly, the data from the radiometer can be used to visualize the incident and reflected short-wave radiation $R_{si}$, the albedo α, the incident and emitted long-wave radiation *L*, and the net radiation *R_n_* (Fig. 4). The two flooding events #2 and #4 are indicated in time. Note that in Fig. 4 values shown for *α* are limited to cases in which the incident short-wave exceeds the mean of the full time series to filter out night (dark) events.

**Fig. 5:**
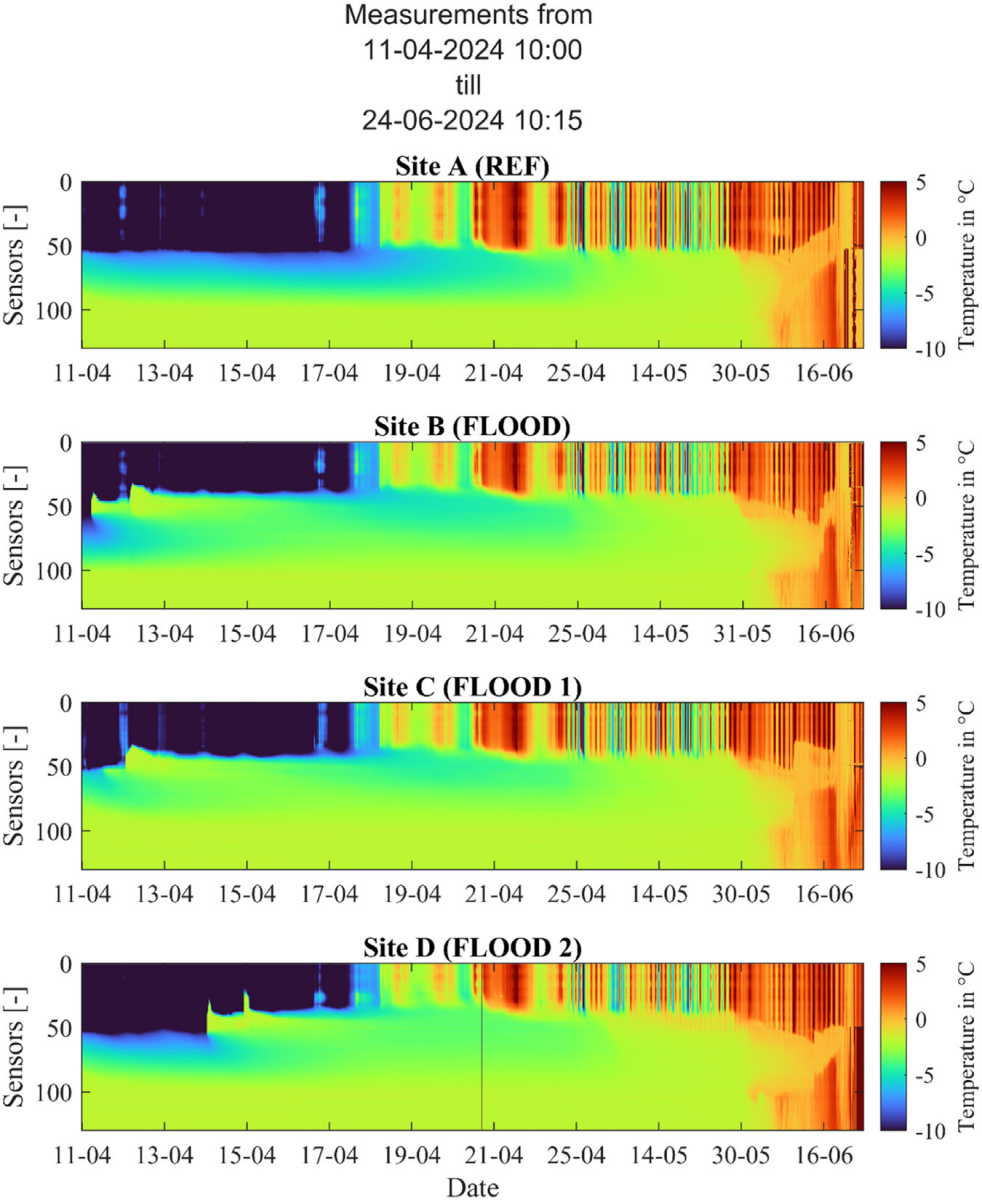
Vertical temperature profiles as measured by the thermistor strings on Site A, B, C and D.

The data from the thermistor string can be used to visualize the vertical temperature profile for the measurement period (Fig. 5).

### 3.2 Ice core sampling

**Fig. 6:**
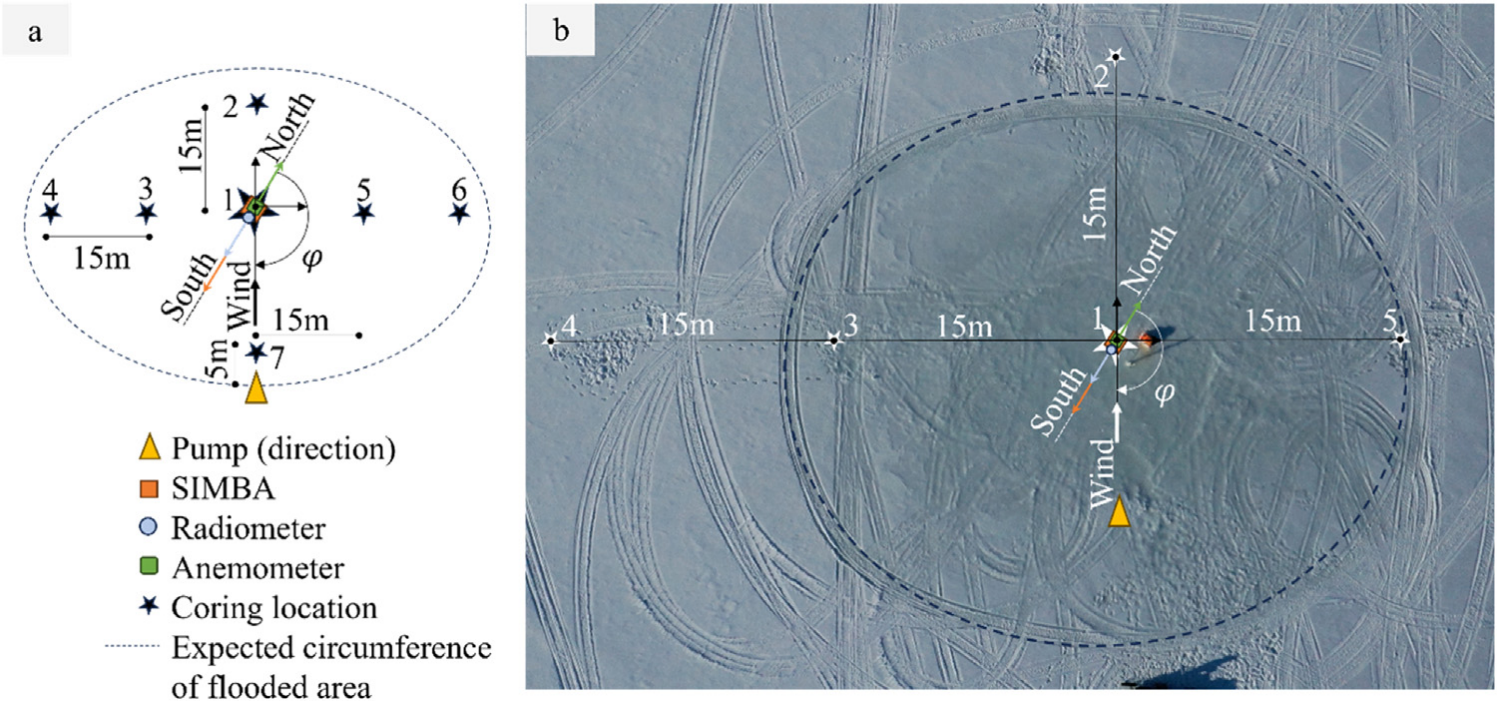
Grid for standardizing the drilling locations per test site (a). Grid applied on Site B in the field. The picture was taken on April 12 (b).

Additional data were generated by taking and analyzing ice core samples on site A, B, and C. A coring grid as shown in Fig. 5 was used in the field to determine the drilling locations. The grid thereby used the measurement station (i.e., location 1) and first pump position on the site as reference. Each test site hosted up to seven planned drilling locations. The reference system used for the data documentation of the sampled ice cores is presented in Fig. 6. A summary of all ice cores taken during the field campaign is given in a meta-data table^7^, which can be found in the data repository [9]. The content of the table is described in Table 6 of the accompanying *readme.pdf* file in the data repository.

**Fig. 7:**
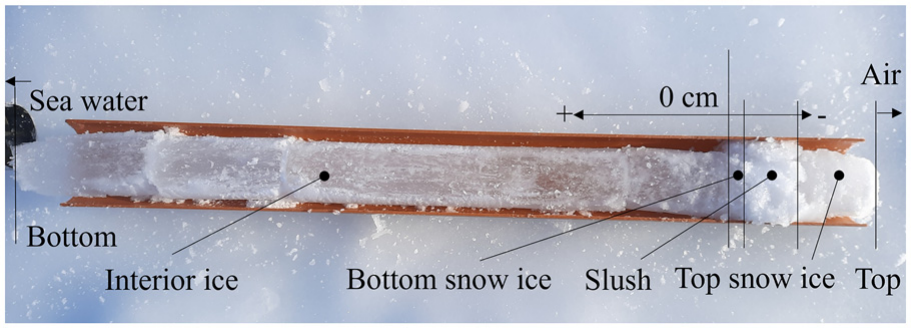
Reference system used during the field campaign. The core was extracted on April 12 after the first flooding on Site B.

Data of temperature measurements along the ice cores are provided as a table^8^, which can be found in the data repository [9]. The content of the table is described in Table 7 of the accompanying *readme.pdf* file in the data repository. Data of density measurements for ice core sections are provided as a table^9^, which can be found in the data repository. The content of the table is described in Table 8 of the accompanying *readme.pdf* file in the data repository.

Data of bulk salinity measurements for ice core sections are provided as a table^10^, which can be found in the data repository. The content of the table is described in Table 8 of the accompanying *readme.pdf* file in the data repository.

**Fig. 8:**
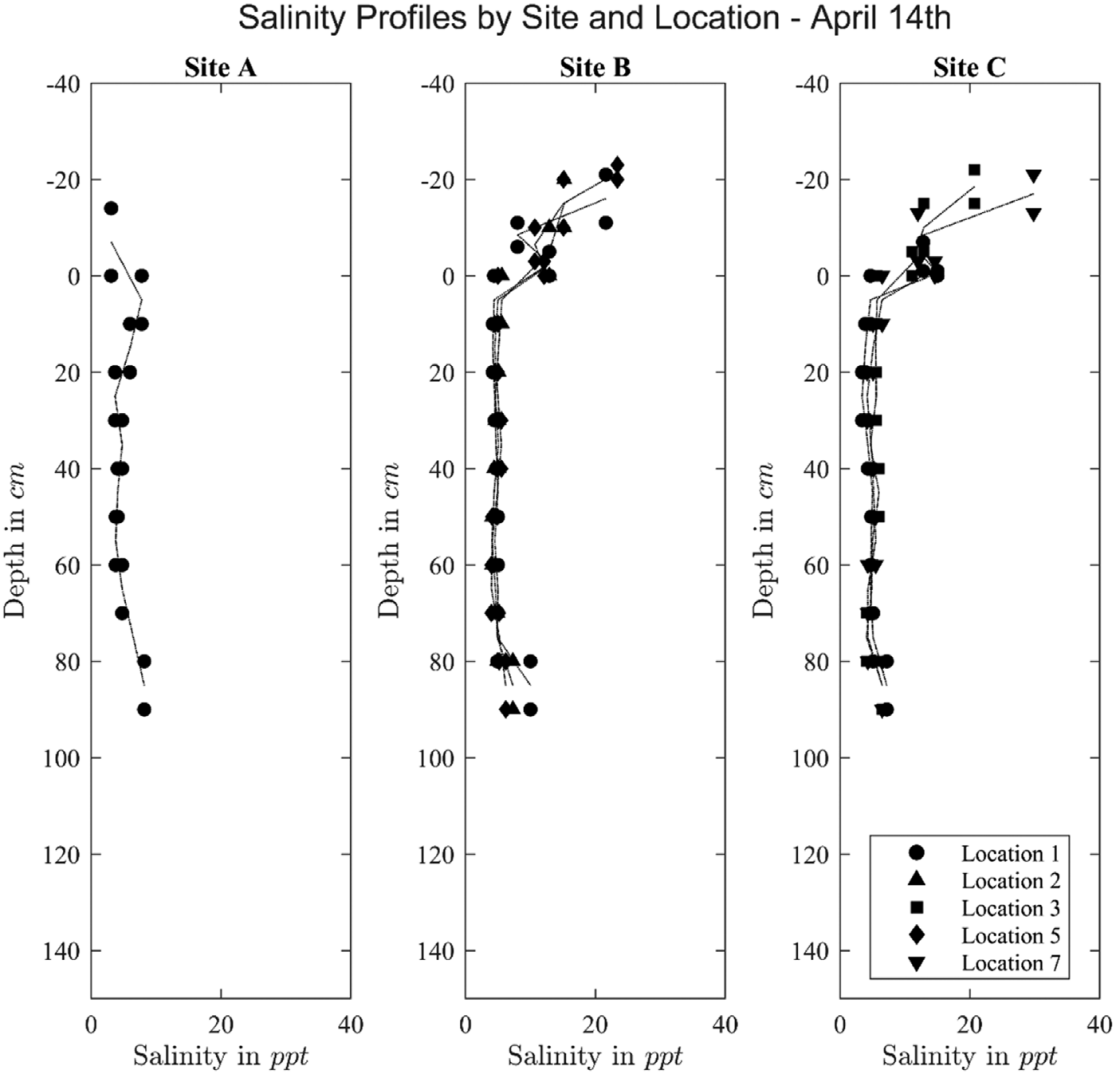
Bulk salinity profiles. Note that the profile shows salinity of ice, snow ice and snow.

Exemplary, bulk salinity profiles are visualized in Fig. 8. In the same manner, temperature and density profiles could be visualized. Note that the same measurement value is shown for the bottom and top position of the investigated ice core section. The line connects the measurement values across the center height of the ice core sections.

### 3.3 Footage

A timelapse video of Vallunden lagoon is provided in *.mp4* format, which was generated using the photographs uploaded in the data repository. The pictures were captured with a sampling period of 15 min over the period of 10 April to 24 June. Pictures used for the timelapse are provided in 15 subfolders (e.g., project-197387-part-1-of-15) in *.jpg* format.

## 4 Experimental Design, Materials and Methods

This section is divided into three subsections. The first subsection explains the experimental design of the measurement stations. The second subsection focuses on the flooding of the test sites. The last subsection focuses on the ice core sampling and methods for measurement of temperature, density, and bulk salinity.

### 4.1 Measurement stations

**Fig. 9:**
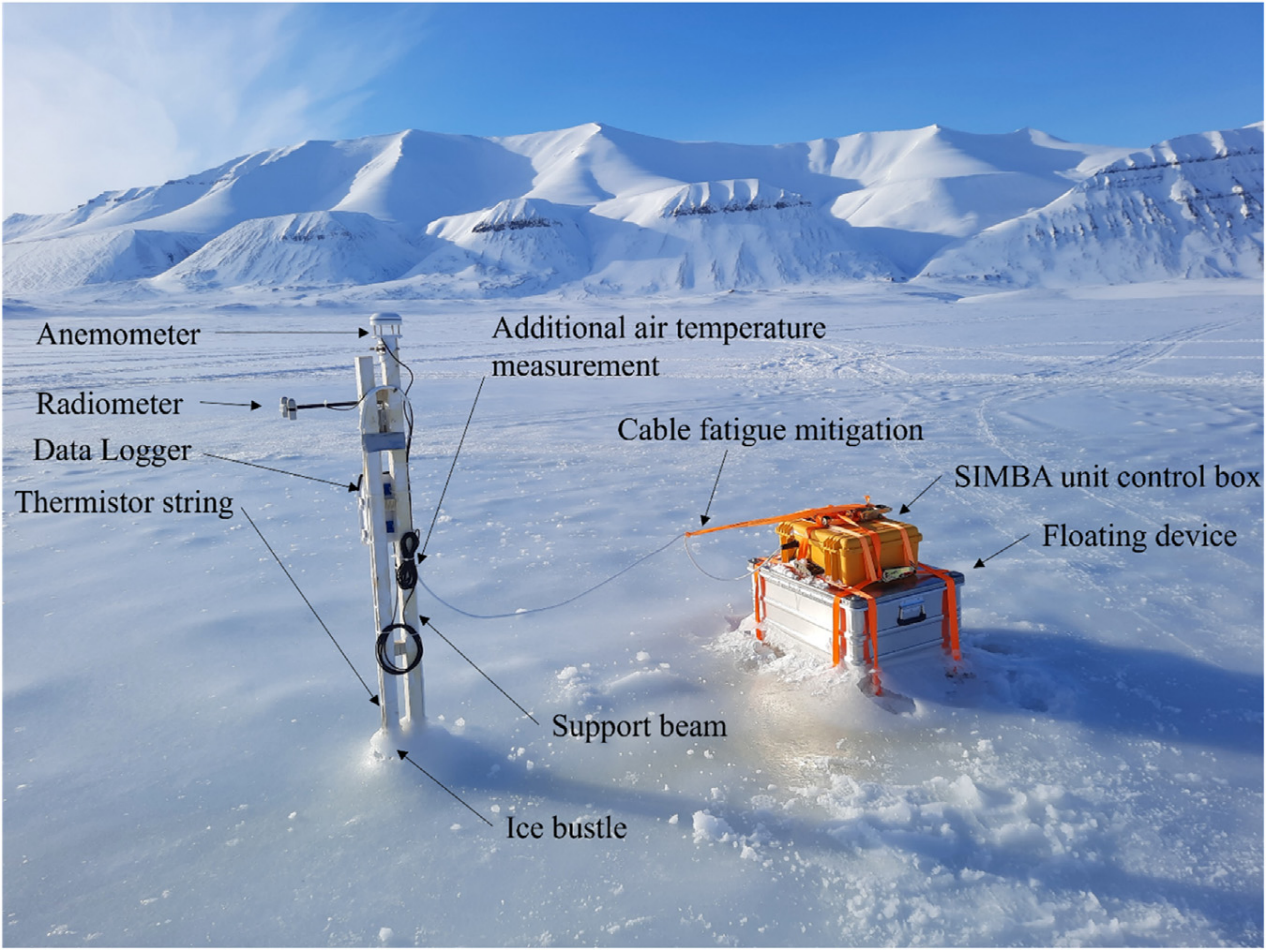
Measurement station in the center of Site B on April 12. Measurement systems as the anemometer, radiometer, thermistor string, data logger and SIMBA unit control box are indicated.

The measurement station installed on site B (see Fig. 9) consisted of a Snow and Ice Mass Balance Apparatus (SIMBA) with a thermistor string, an anemometer, a radiometer, and a data logger. The SIMBA provided an integral satellite modem and GPS (for recovery). The string was attached to a wooden guiding beam which was fastened to a wooden support beam. The last meter of the support beam was milled, resulting in a round profile which could be installed in a pre-drilled 2'' hole in the ice. Overnight the snow was kept removed from the installation square (uncovered ice surface) to allow for faster freezing of the thermistor string into the ice on Site A, C, and D (see Fig. 9). Note that the thermistor string on Site B was installed three weeks earlier than those on Site A, C, and D.

**Fig. 10:**
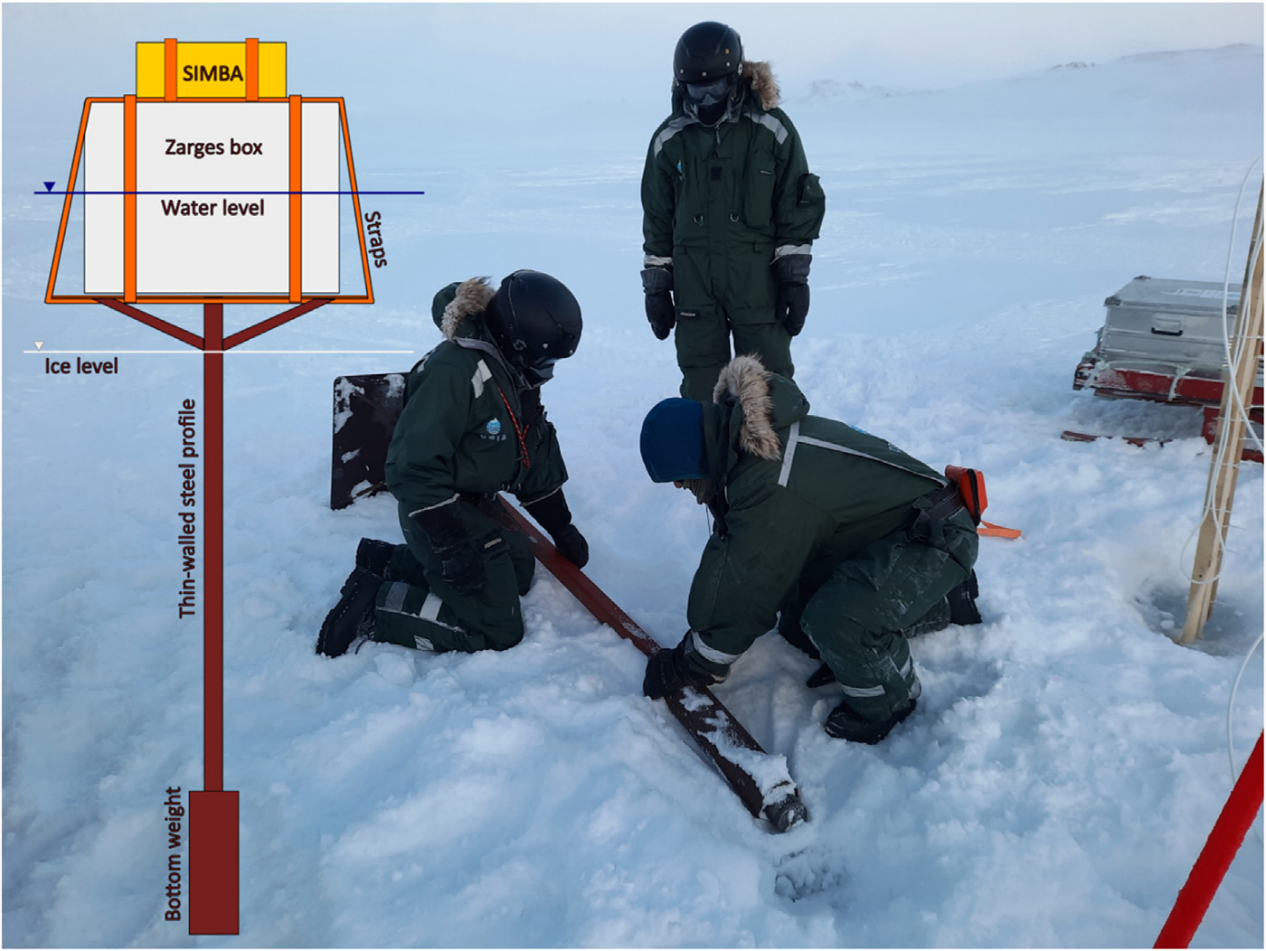
Technical sketch and installation of the floating device, increasing the probability of a successful recovery of the SIMBA control box and attached wooden pile in the summer.

Once the sensors were installed, the SIMBA unit control box was attached to a floating device designed to keep the control box afloat once the ice would start melting, facilitating recovery and allowing the system to be reused. The floating device consisted of a floating platform made from a thin steel plate and a thin-walled steel profile as can be seen in Fig. 10. An aluminum box, which was prefilled with Styrofoam, was attached to the platform using wide straps. The control box of the SIMBA was attached to the aluminum box using thin straps.

The cable connecting the thermistor string and SIMBA unit control box was tensioned to minimize the risk of fatigue damage caused by the cable galloping in the wind. On the next morning, after the measurements showed that the string had frozen into place, snow was filled up to the original snow layer depth. The same experimental design, as shown in Fig. 10, was used for the measurement stations installed on Site A, C, and D. However, the measurement station on Site A had no anemometer installed; the measurement station on Site C and D had neither anemometer nor radiometer installed; and the SIMBA unit on Site D was not attached to a floating device. The measurement stations provided continuous data as explained above.

### 4.2 Flooding of test sites

**Fig. 11:**
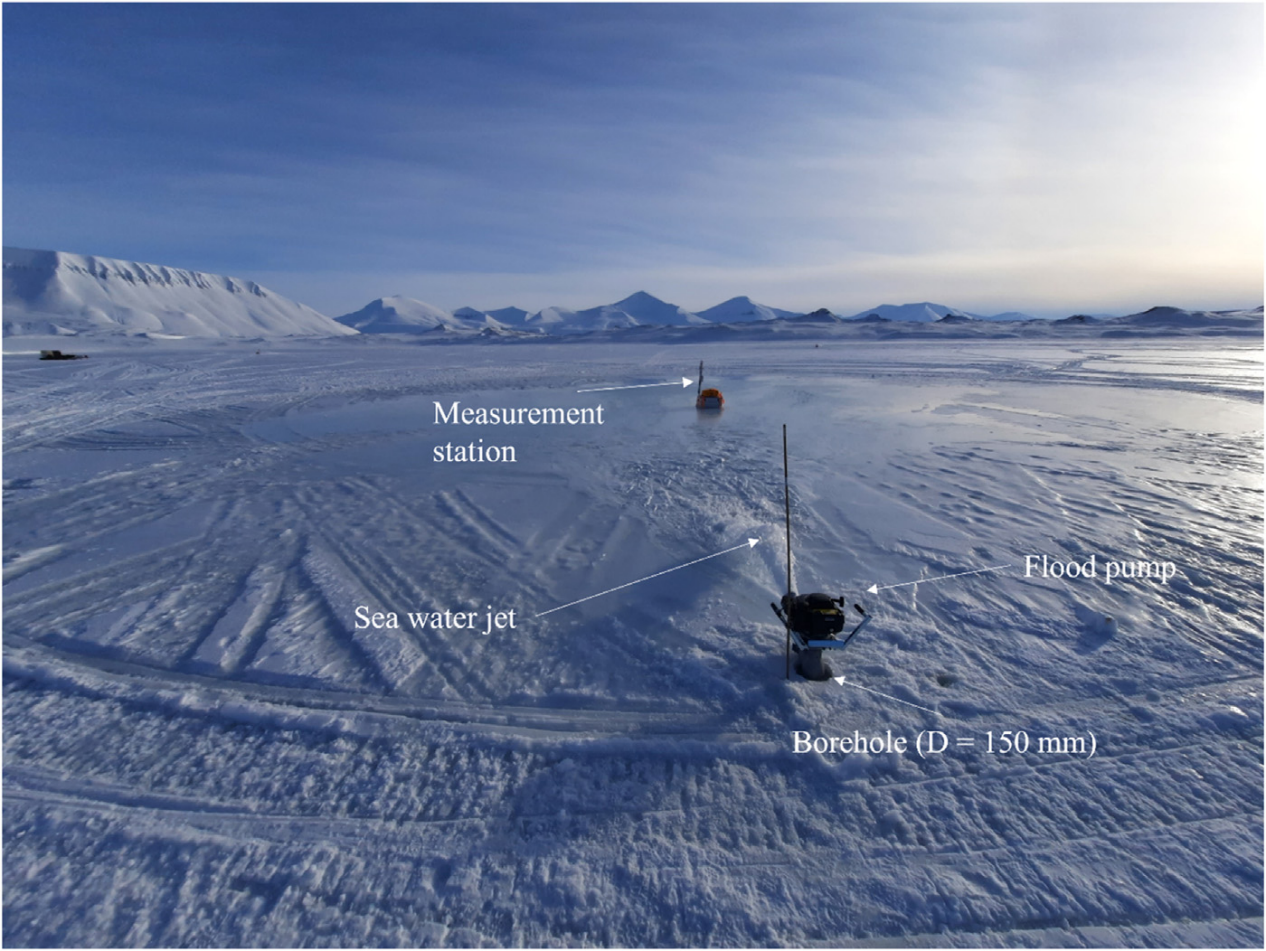
Flood pump pumping water at Site B on April 12.

**Fig. 12:**
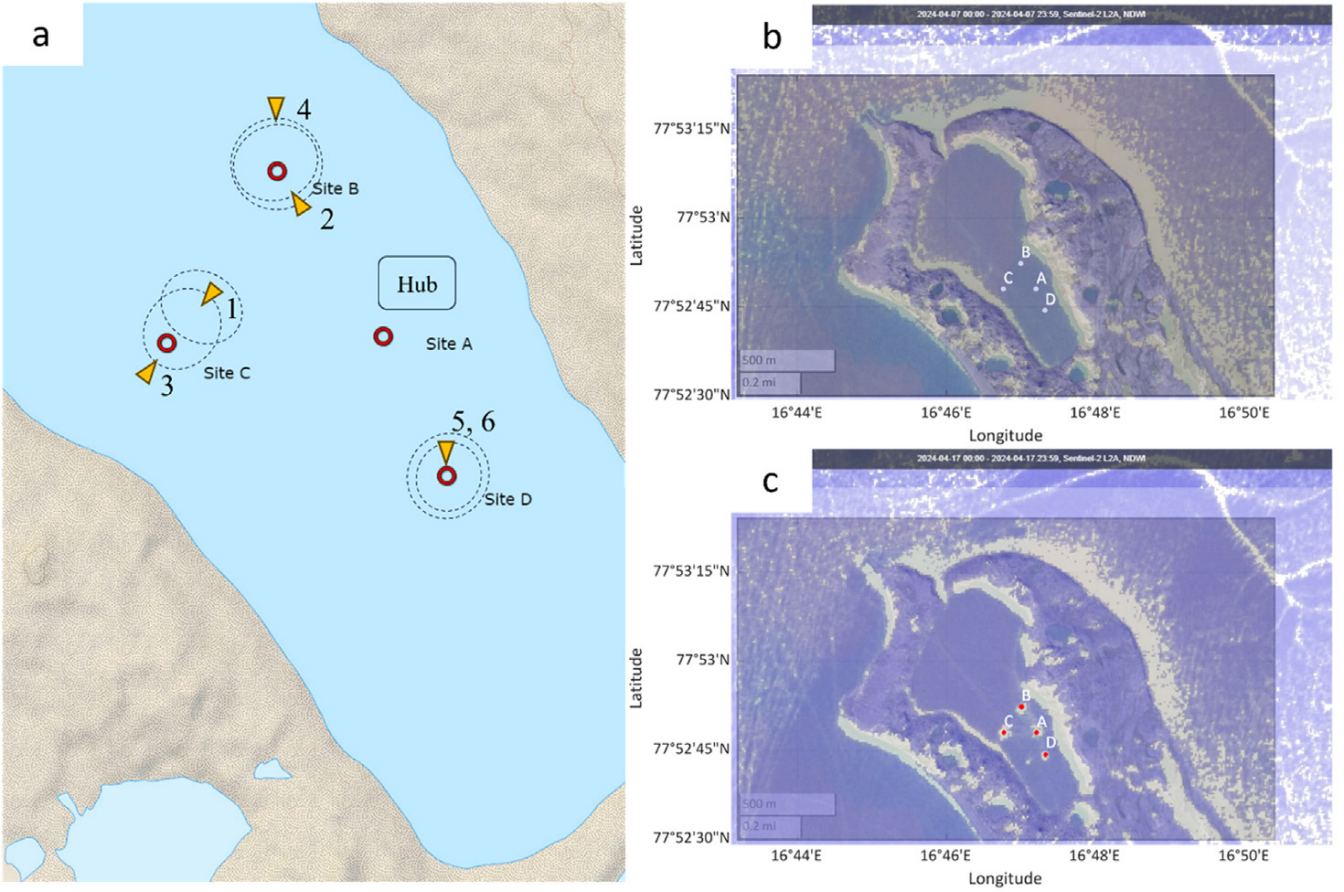
Visualization of pum position, direction and flooded area (a). Satellite footage from the Sentinel L2A satellite using the normalized difference water index layer indicates flooded areas before flooding (April 7-b) and after flooding (April 17-c).

A 150 mm ice auger was used to drill a borehole into the ice for installation of the flood pump. Brash ice and larger ice fragments floating in the borehole were removed with a sieve, before the pump was installed. Once installed, a sea water jet stream would start to flood the snow cover (see Fig. 11). The motor was supposedly running on full power for all flooding events. The jet flooded the area in close vicinity to the pump quickly. Soon after, the water started to flood the snow layer further away from the pump. A bamboo stick was used to constrain any rotation of the flood pump. Specific pump positions and affected areas are visualized in Fig. 12 and further details on the flooding events are listed in Table 2.

**Table 2:** Specifications of flooding events.

Order	Site	Date	Direction	Start-Refuel-End	Distance to SIMBA	Affected Area⁎
1	C	11.04.2024	SW	16:00–17:30	22 m	–
2	B	11.04.2024	NNW	17:30–18:45	10 m	–
3	C	12.04.2024	NE	14:53–16:02–17:47–18:53	15 m	3300 m^2^
4	B	12.04.2024	S	17:10–19:00†	18 m	2400 m^2^
5	D	14.04.2024	S	16:05–17:37	unknown	–
6	D	15.04.2024	S	14:35-unknown	unknown	1600 m^2^

⁎ Estimation based on the satellite data shown in Figure 11-c (assuming circular shape of flooded areas).

† One diary entry indicates that the pump was not running at full throttle.

**Fig. 13:**
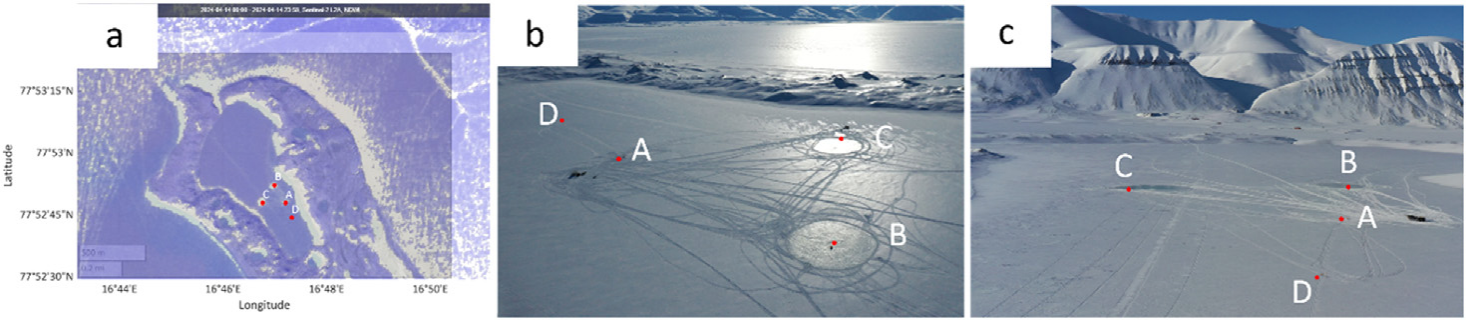
Comparing satellite (14.04-a) with aerial footage (12.04-b) verifies that the white spots on Site B and C are related to the flooding, while the white spot on site A is caused by changes of the snow surface (c).

Here, the affected area (i.e. affected by flooding) was estimated based on the satellite data shown in Fig. 12-c assuming circular shape of flooded areas. The usage of the satellite data for flooded area estimation can be verified when comparing satellite data of April 14 with aerial footage that was taken two days earlier (12.04. – Fig. 13). The white spots shown by the satellite on Site A are attributed to changes of the snow surface in close vicinity of the central hub (e.g. caused by scooter driving or walking). Note that due to the deterioration of the weather conditions on April 15, no aerial footage could be taken from Site D after flooding. The white spot identified by the satellite to the west of Site D in Fig. 12-c is attributed to a test pumping.

Note that the first flooding event as shown in Fig. 12-a did not lead to the measurement station being flooded superficially. This is why the pump was installed closer to the measurement station in follow-up flooding events. The fuel typically lasted 1 to 2 hrs before the pump had to be refueled or deinstalled. The deinstallation of the pump was complicated when the water had surrounded the pump. Removing the pump in such scenarios could lead to a drainage of the hydraulic water reservoir when the hole was not closed in time. Thus, the drilling hole had to be closed with compacted snow or similar after removal of the pump. Once deinstalled, the pump was stored inside a heated cabin thawing ice covered mechanical parts.

**Fig. 14:**
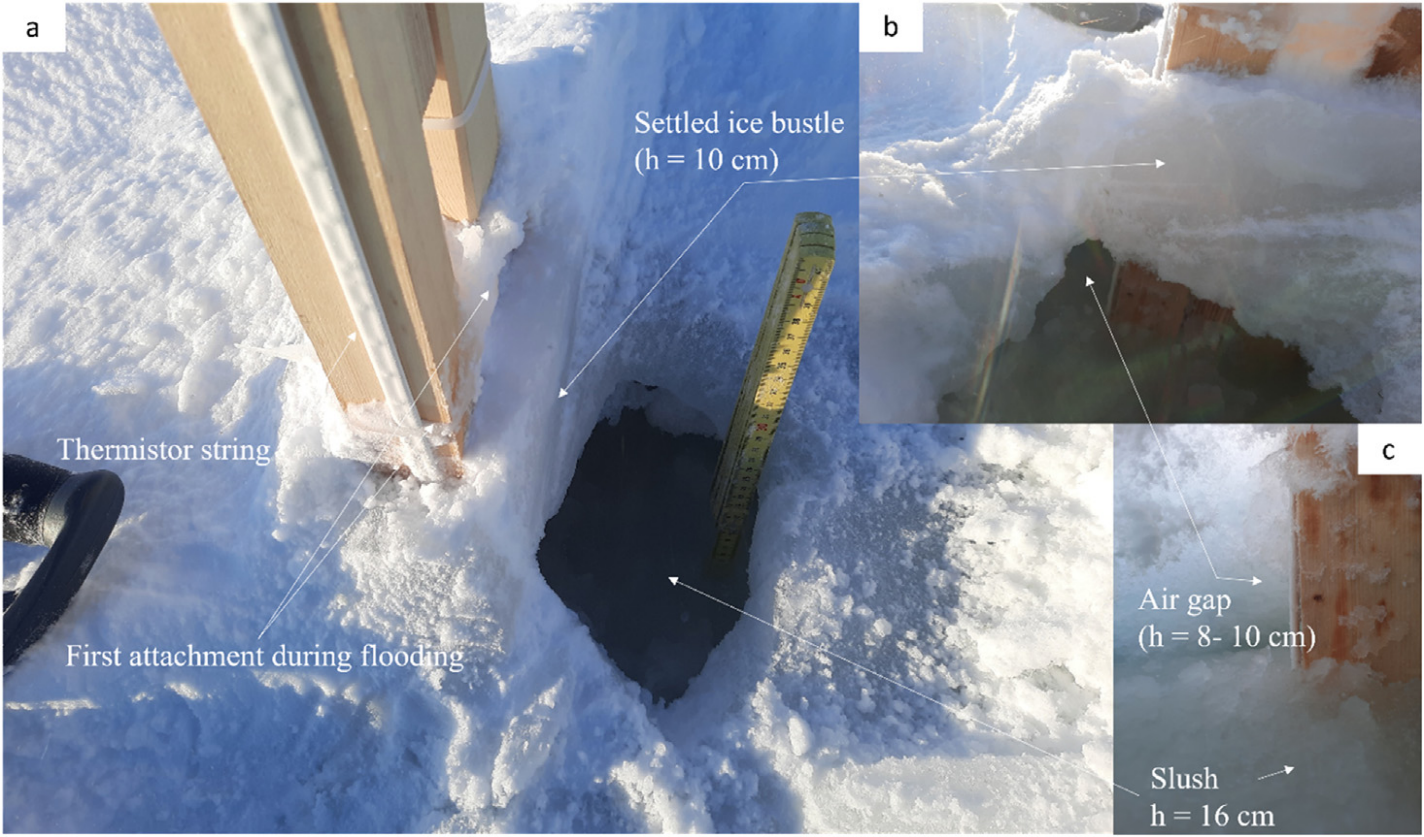
A ‘cave’ and ice bustle were forming in close vicinity to the measurement station on Site C (a). The ice bustle was attached to the measurement station and could increase the local ice thickness as measured by the thermistor string (b). An air gap between the slush and ice bustle was observed (c).

On the morning of April 12, it was noticed that ice bustles had formed at the wooden support beam (see Fig. 10). As it was assumed that the ice bustle could affect the measurement of the thermistor string by projecting a ‘fake’ consolidated snow ice thickness, the ice bustles were investigated on April 13 at 18:33 local time. Thereby it was discovered that a ‘cave’ had formed below the ice bustle on Site C (see Fig. 14). The cave consisted of a 8 to 10 cm air gap (see Fig. 14-c) below the ice bustle which had a height of roughly 10 cm (see Fig. 14-b). A 16 cm thick slush layer was measured below the air gap (see Fig. 14-c). The exposure of the thermistor strings to local effects, like the ‘cave’ forming, underlines the importance of in-situ ice core sampling which are explained in the next section. The ice bustle on Site B was smaller and only a small slush layer without air gap was found when opening the top ice layer at the site of the measurement station.

### 4.3 In-situ ice coring and ice core measurements

Ice core drilling locations were determined using the grid presented in Fig. 6. Note that the distance between the pump and the measurement station varied as documented in Table 2. The grid was applied for the flooding events #2 and #3 for Site B and C, respectively. For better comparability the grid on Site A (no flooding) was applied in the same direction as for Site B.

At least two ice cores have been taken on each drilling location. Two ice core systems have been used to allow parallel ice core sampling. Thus, on Site B and C, a Kovacs Mark System III was used; on Site A, a Kovacs Mark System II was used. Both systems were applied using an electrical Hitachi drill. Only in cases of low battery, a fuel-powered Stiehl engine in combination with personal noise protection gear was used. The core barrel was supposed to be inserted near-vertically into the ice during drilling. The borehole was closed after coring.

The first of the two ice cores drilled at each location was immediately transported to the temperature measurement station to determine a temperature profile along the length of the core. The same core was then used to determine a density profile along the length of the core. The second ice core was used for bulk salinity measurements, resulting in a bulk salinity profile along the length of the core. The three measurements are explained in further detail in the next three subsections.

#### 4.3.1 Temperature profile measurements

**Fig. 15:**
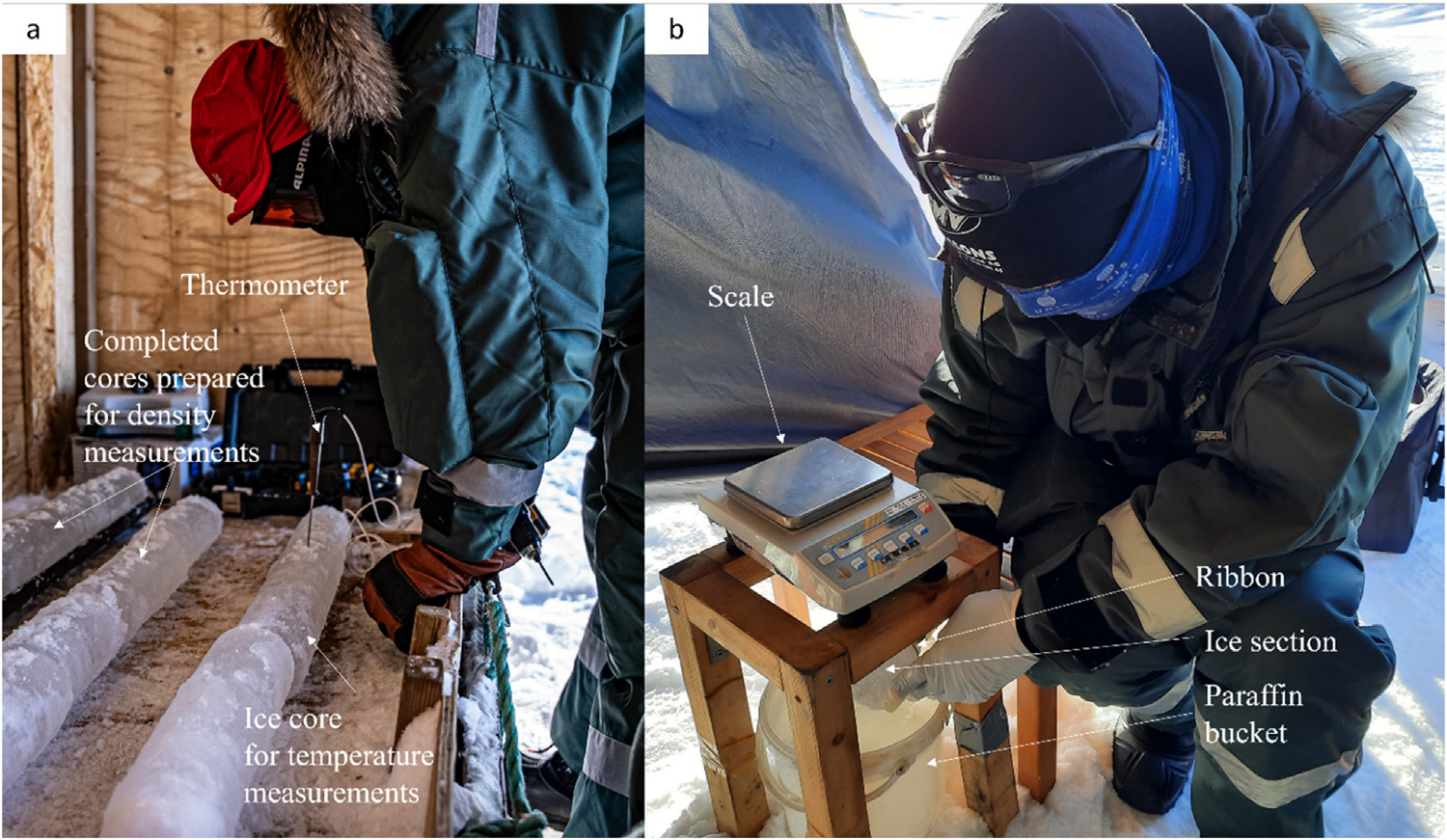
Ice core temperature measurement along the core (a). Ice density measurement for single ice sections (b).

One ice core was transported immediately to the measurement station to minimize the influence of air temperature on the measurement of the ice core temperature. An electrical drill was used to drill a hole into the center of the ice core. Next, the probe of the thermometer was inserted. The temperature reached a steady-state temperature after roughly 5–10 s. This steady-state temperature was documented. The procedure was repeated every 5 cm along the ice core (Fig. 15-a).

#### 4.3.2 Density profile measurements

After measuring the time-critical temperature along the ice core, the core was cut into pieces of 10 cm length using a hand saw. The freshly cut sections were cleaned by scraping them with a knife or glove ensuring there was no residue left. Masses of the prepared ice core sections were determined with a high precision scale. Next, the submerged mass of the ice section was measured. Therefore, a thin ribbon was attached to the ice section. The ribbon was hinged to a small hook beneath the scale which was connected to the weighting mechanism. Once hinged, the ice section was submerged in a bucket of paraffin below the scale (see Fig. 15-b). The submerged mass was documented once the ice section was fully submerged but still floating (i.e., the ice section had no contact to the bottom of the bucket). The temperature of the paraffin was measured at least once a day to define the temperature-dependent density of the fluid during the postprocessing. The density of the ice section $\rho_{ice}$ can be determined using the submerged mass $m_{sub}$, the ice section mass $m_{dry}$, and the density of the paraffin $\rho_{p}$:

(1) $$\rho_{ice}=\frac{m_{dry}\cdot \rho_{p}}{m_{dry}-m_{sub}}$$

#### 4.3.3 Bulk salinity profile measurements

The second ice core sampled in the field was used to create a bulk salinity profile along the ice core. The ice core was cut into pieces of 10 cm length, double-bagged and labelled. The double-bagged probes were transported to a heated cabin. Once melted, the solution was stirred up 2–5 times before documenting the bulk salinity and temperature of the solution with a conductivity meter.

### 4.4 Footage

**Fig. 16:**
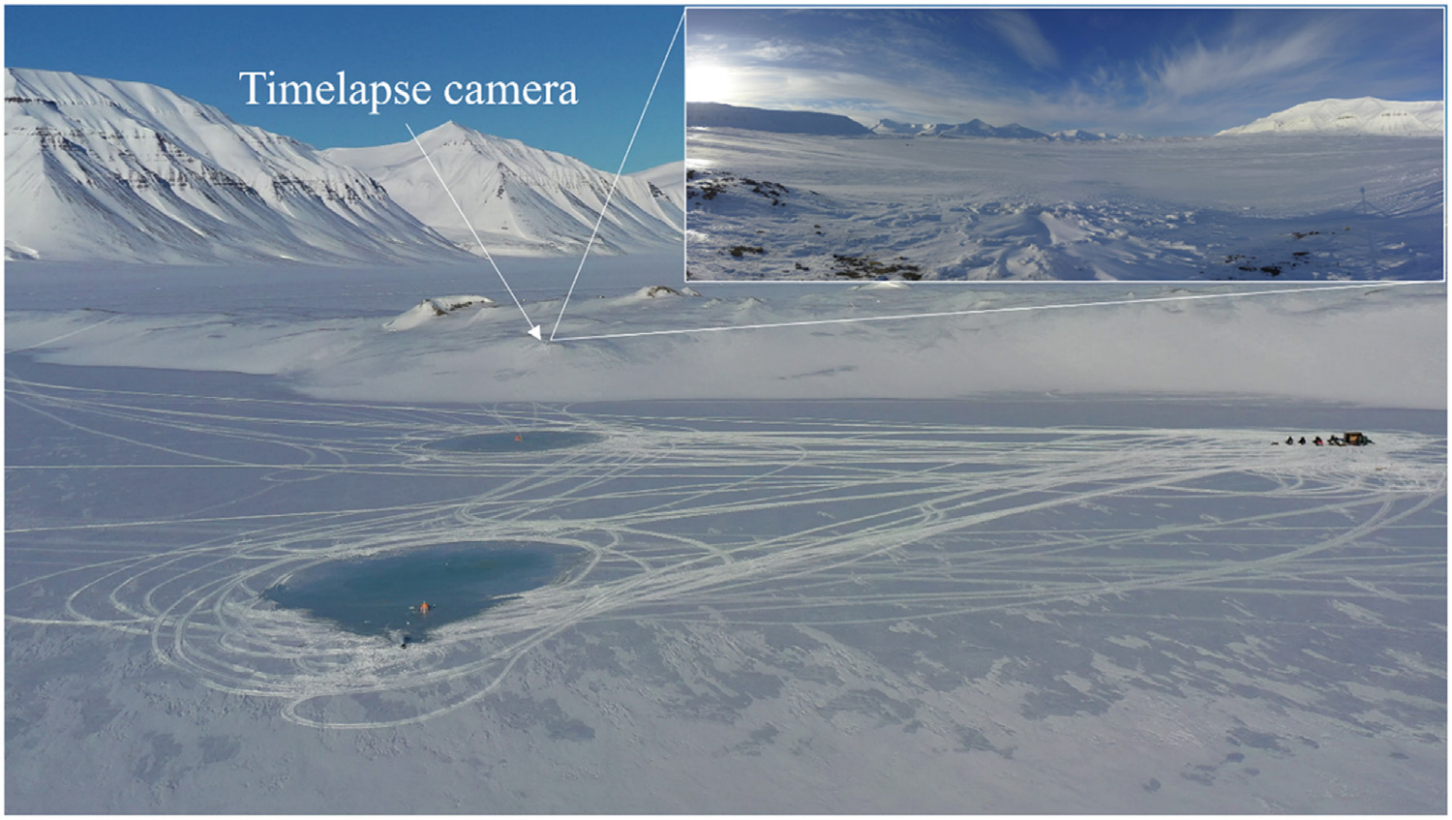
Picture taken by the drone on April 12 at 8:50 and ten minutes later by the timelapse camera.

A stationary timelapse camera aiming at the test sites was used to record the installation, flooding and melting period. The position and view of the camera is shown in Fig. 16. Both the raw pictures and a time lapse video of the stationary pictures are provided in the data repository.

## Limitations

Data collected during the temperature, bulk salinity, and density measurements of ice core sections for negative coordinates (i.e., flooded layer) are difficult to interpret. One reason was that a slush layer between the flooded and initial ice surface formed (e.g., Fig. 14) and drained when the core was extracted. Thus, data recorded in the flooded top layer could not always be referred to a specific position on the axis and data on the slush is limited. Also, a snow ice section could fail when it was tried to drill a hole for temperature measurements or to attach the ribbon during the density measurements. Analysis of the snow ice layer must thus be used keeping this uncertainty in mind and should be compared to additional comments given in the meta-data matrix (i.e., Core_matrix.xlsx) giving further description of the top ice layer. Sampling frequencies have been changed on two ad-hoc instances which has to be considered in data visualization. The paraffin was changed during the last test day. While the original paraffin was specially cleaned, the substitute was raw paraffin which can affect the accuracy of the density measurements. Measurements provided by the thermistor string on Site C are affected by the formation of an ice bustle, most likely caused by a hydraulic water reservoir forming during flooding (see Fig. 9). The anemometer on Site B indicated that the pole started to tilt during the melting phase. The tilt might have affected the radiometer measurements which should be analyzed carefully. The stationary camera shows a warped picture since the May 10 from 20:44 onwards. Data collected during the melting of the ice might be affected by (local) drain channel formation along the thermistor string, and by a tilt of the wooden beam (i.e. tilt of radiometer and anemometer). Due to a limited period of in-situ ice coring, the spatial variability of the measurements are limited.

## Ethics Statement

The present study meets the ethical requirements.

## Credit Author Statement

**Tim C. Hammer:** Conceptualization, Methodology, Validation, Investigation, Data Curation, Writing - Original Draft, Visualization, Supervision, Project administration; **Laurina Leuntje van Dijke**: Conceptualization, Methodology, Validation, Investigation, Data Curation, Project administration, Funding acquisition; **Aleksey Shestov**: Conceptualization, Methodology, Validation, Investigation, Resources, Supervision, Project administration; **Fonger Ypma:** Investigation, Resources, Funding acquisition; **Tom Meijeraan:** Investigation, Resources, Funding acquisition; **Hayo Hendrikse:** Conceptualization, Investigation, Writing – Review & Editing, Resources, Project administration, Funding acquisition.

## Data Availability

4TU.ResearchDataData from Field Experiments on Sea Ice Restoration by Artificial Flooding (Original data)..

## Footnotes

1 reflar0104td_2024-06-25_07-27-47.csv

2 reflar0101td_2024-06-25_07-27-40.csv

3 reflar0103td_2024-06-25_07-27-45.csv

4 reflar0102td_2024-06-25_07-27-42.csv

5 Site_B(z6-25195)-1719296946.mat

6 Site_A(z6-25201)-1719296946.mat

7 Core_matrix.xlsx

8 Tempertaure_measurements_all.xlsx

9 Density_measurements_all.xlsx

10 Bulk salinity_measuremts_all.xlsx
